# Heme Oxygenase-1 Induction by Cobalt Protoporphyrin Ameliorates Cholestatic Liver Disease in a Xenobiotic-Induced Murine Model

**DOI:** 10.3390/ijms22158253

**Published:** 2021-07-31

**Authors:** Jung-Yeon Kim, Yongmin Choi, Jaechan Leem, Jeong Eun Song

**Affiliations:** 1 Department of Immunology, School of Medicine, Catholic University of Daegu, Daegu 42472, Korea; jy1118@cu.ac.kr; 2 Department of Rehabilitation Medicine, School of Medicine, Keimyung University, Daegu 42601, Korea; ymchoi@dsmc.or.kr; 3 Department of Internal Medicine, School of Medicine, Catholic University of Daegu, Daegu 42472, Korea

**Keywords:** heme oxygenase-1, cholestatic liver disease, oxidative stress, apoptosis, inflammation, fibrosis

## Abstract

Cholestatic liver diseases can progress to end-stage liver disease and reduce patients’ quality of life. Although their underlying mechanisms are still incompletely elucidated, oxidative stress is considered to be a key contributor to these diseases. Heme oxygenase-1 (HO-1) is a cytoprotective enzyme that displays antioxidant action. It has been found that this enzyme plays a protective role against various inflammatory diseases. However, the role of HO-1 in cholestatic liver diseases has not yet been investigated. Here, we examined whether pharmacological induction of HO-1 by cobalt protoporphyrin (CoPP) ameliorates cholestatic liver injury. To this end, a murine model of 3,5-diethoxycarbonyl-1,4-dihydrocollidine (DDC) diet feeding was used. Administration of CoPP ameliorated liver damage and cholestasis with HO-1 upregulation in DDC diet-fed mice. Induction of HO-1 by CoPP suppressed the DDC diet-induced oxidative stress and hepatocyte apoptosis. In addition, CoPP attenuated cytokine production and inflammatory cell infiltration. Furthermore, deposition of the extracellular matrix and expression of fibrosis-related genes after DDC feeding were also decreased by CoPP. HO-1 induction decreased the number of myofibroblasts and inhibited the transforming growth factor-β pathway. Altogether, these data suggest that the pharmacological induction of HO-1 ameliorates cholestatic liver disease by suppressing oxidative stress, hepatocyte apoptosis, and inflammation.

## 1. Introduction

Cholestatic liver diseases are characterized by cholestasis, bile duct injury, and fibrosis [1]. Despite largely expanded research efforts in recent decades, cholestatic liver diseases still remain as the main contributors to liver-associated morbidity and mortality [2]. Liver transplantation is the mainstay therapy for these diseases, and there are no effective medical therapies. Although the pathogenesis of cholestatic liver diseases remains incompletely understood, oxidative stress is considered to be one of the important pathogenic factors [3,4]. Previous human studies have reported that oxidative stress was increased in patients suffering from cholestatic liver diseases [5,6]. Animals with cholestatic liver injury also exhibited increased expression of oxidative stress markers in the liver [7,8]. Recent studies have shown that various compounds alleviated cholestatic liver injury via inhibiting oxidative stress in animals [9,10,11]. Therefore, suppression of oxidative stress might be a useful therapeutic strategy for cholestatic liver diseases.

Heme oxygenase (HO) is an enzyme that catalyzes heme degradation to generate biliverdin, free iron, and carbon monoxide [12]. To date, two main isoforms of this enzyme—HO-1 and HO-2—have been identified. Among them, HO-1 is a stress-inducible enzyme that displays antioxidant activity [13]. Its expression is maintained at low levels under basal conditions, but is largely increased in response to various pathological stresses [13]. Byproducts generated by HO-1 exert potent antioxidative and anti-inflammatory actions [12]. Thus, induction of HO-1 may be a potential therapeutic strategy for human diseases. Growing evidence suggests that the pharmacological induction of HO-1 by cobalt protoporphyrin (CoPP) or hemin ameliorates various inflammatory diseases [12,13,14]. However, the role of HO-1 in cholestatic liver injury remains incompletely understood.

The 3,5-diethoxycarbonyl-1,4-dihydrocollidine (DDC) diet model is widely used for exploring the pathogenesis of, and therapeutic approaches to, cholestatic liver diseases [15,16]. DDC feeding in mice reproduces the major histological characteristics of human cholestatic liver disease—such as remodeling of biliary compartments, periductal fibrosis, and inflammatory cell infiltration—by increasing the formation of intraductal porphyrin plugs [15]. In the present study, we examined whether the induction of HO-1 by CoPP could ameliorate DDC-diet-induced cholestatic liver injury, and investigated the specific mechanisms.

## 2. Results

### 2.1. Administration of CoPP Ameliorated Liver Damage and Cholestasis in DDC-Fed Mice

**Figure 1:**
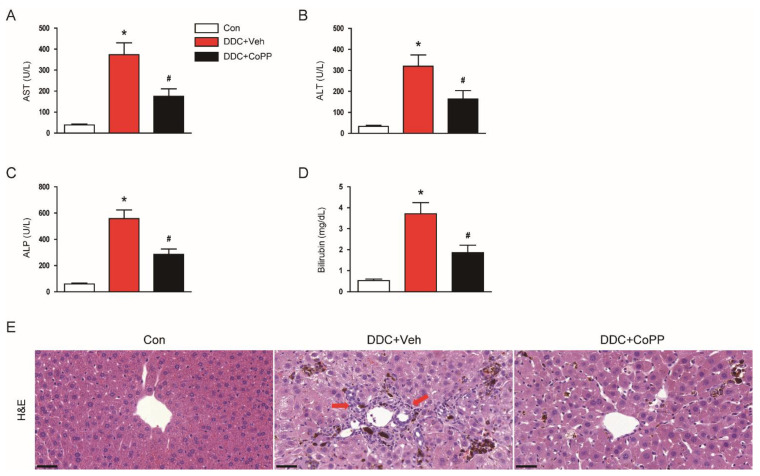
Biochemical parameters and histological changes in the liver, in all study groups. C57BL/6N mice were fed with a diet containing 0.1% 3,5-diethoxycarbonyl-1,4-dihydrocollidine (DDC), and were intraperitoneally injected with cobalt protoporphyrin (CoPP) twice a week for 4 weeks. (**A**) Serum aspartate aminotransferase (AST) levels. (**B**) Serum alanine aminotransferase (ALT) levels. (**C**) Serum alkaline phosphatase (ALP) levels. (**D**) Serum total bilirubin levels. (**E**) Hematoxylin and eosin (H&E) staining of liver tissues. Red arrows indicate immune infiltrates. Scale bar = 40 μm. n = 8 per group. * *p* < 0.05 vs. the control group (Con). ^#^ *p* < 0.05 vs. the DDC-fed group (DDC + Veh).

DDC feeding led to elevated serum levels of aspartate aminotransferase (AST; Figure 1A) and alanine aminotransferase (ALT; Figure 1B)—indicators of hepatocyte injury—in mice. Serum markers of cholestasis, alkaline phosphatase (ALP; Figure 1C), and total bilirubin (Figure 1D) were also highly elevated in DDC-fed mice compared to control mice. However, the administration of CoPP significantly inhibited the DDC-diet-induced liver damage and cholestasis (Figure 1A–D).

Hematoxylin and eosin staining of liver tissues revealed that the DDC-fed mice exhibited histological injuries, such as deposition of pigment plugs in small bile ducts and immune infiltrates (Figure 1E). These histological alterations were also significantly attenuated by CoPP (Figure 1E).

### 2.2. Induction of HO-1 by CoPP Attenuated Oxidative Stress in DDC-Fed Mice

**Figure 2:**
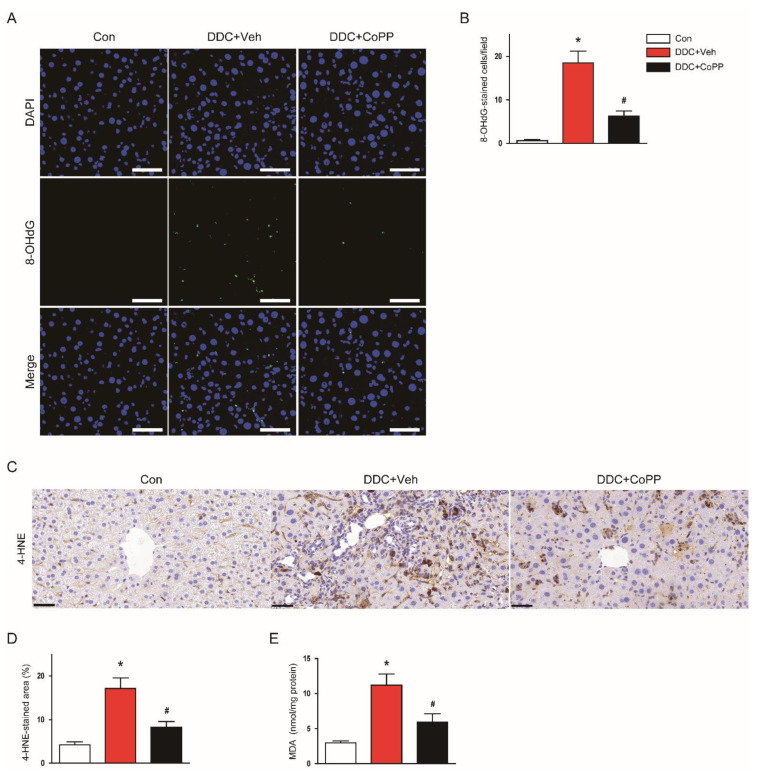
Effect of CoPP on DDC-diet-induced hepatic oxidative stress. (**A**) Immunofluorescent staining of liver tissues for 8-hydroxy-2′-deoxyguanosine (8-OHdG). Scale bar = 40 μm. Nuclei were counterstained with 4′, 6-diamidino-2-phenylindole (DAPI). (**B**) Number of 8-OHdG-stained cells/field. (**C**) Immunohistochemical staining of liver tissues for 4-hydroxynonenal (4-HNE). Scale bar = 100 μm. (**D**) Percentage of 4-HNE-stained area. (**E**) Malondialdehyde (MDA) levels in liver tissues. n = 8 per group. * *p* < 0.05 vs. Con. ^#^ *p* < 0.05 vs. DDC + Veh.

Oxidative stress plays a key role in cholestatic liver diseases [3,4]. To evaluate the effect of CoPP on oxidative stress, we performed immunofluorescent staining for 8-hydroxy-2′-deoxyguanosine (8-OHdG). This molecule is a widely used marker of oxidative-stress-induced DNA damage [17]. DDC-fed mice exhibited an increase in the number of 8-OHdG-stained cells compared to control mice (Figure 2A,B). Immunohistochemical staining for 4-hydroxynonenal (4-HNE)—a major product of lipid peroxidation [18]—revealed that the percentage of the 4-HNE-stained area was increased after DDC feeding (Figure 2C,D). Hepatic levels of malondialdehyde (MDA)—another marker of lipid peroxidation [19]—were also increased (Figure 2E). However, the DDC-diet-induced oxidative stress was significantly suppressed by CoPP (Figure 2A–E).

**Figure 3:**
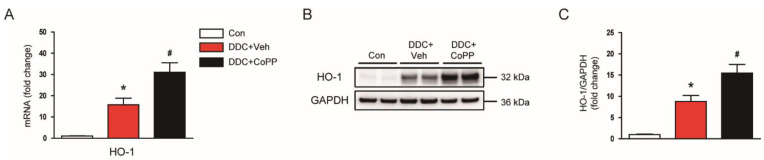
Effect of CoPP on heme oxygenase-1 (HO-1) expression in DDC-fed mice. (**A**) The mRNA levels of HO-1 in liver tissues. (**B**) Western blotting of HO-1 in liver tissues. (**C**) Quantification of Western blot for HO-1. Glyceraldehyde-3-phosphate dehydrogenase (GAPDH) was used as a loading control. n = 8 per group. * *p* < 0.05 vs. Con. ^#^ *p* < 0.05 vs. DDC + Veh.

HO-1 is a cytoprotective enzyme that has antioxidant properties [12]. Its expression is largely increased in response to a variety of pathological stresses. DDC feeding resulted in increased levels of HO-1 mRNA (Figure 3A) and proteins (Figure 3B,C) in the liver. Interestingly, administration of CoPP further enhanced the expression of HO-1 (Figure 3A–C). Altogether, these results indicate that CoPP inhibited DDC-diet-induced oxidative stress, along with enhancement of HO-1 expression.

### 2.3. Induction of HO-1 by CoPP Inhibited Apoptotic Cell Death in DDC-Fed Mice

**Figure 4:**
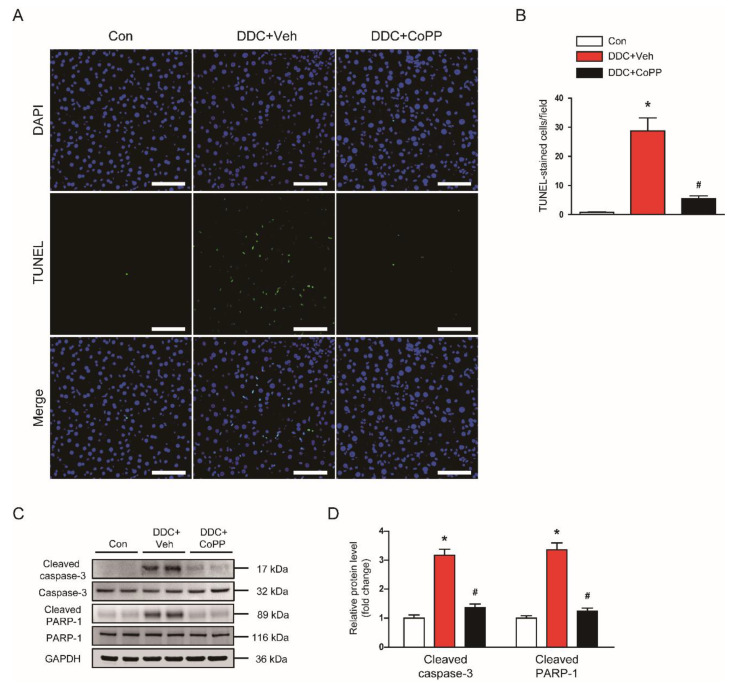
Effect of CoPP on apoptosis in DDC-fed mice. (**A**) TdT-mediated dUTP nick end labeling (TUNEL) assay on liver tissues. Scale bar = 50 μm. (**B**) Number of TUNEL-stained cells. (**C**) Western blotting of cleaved caspase-3 and cleaved poly(ADP-ribose) polymerase-1 (PARP-1) in liver tissues. (**D**) Quantification of Western blots for cleaved caspase-3 and cleaved PARP-1. n = 8 per group. * *p* < 0.05 vs. Con. ^#^ *p* < 0.05 vs. DDC + Veh.

Apoptotic death of hepatocytes is a crucial event in cholestatic liver diseases, leading to inflammatory responses and fibrosis [20]. Oxidative stress can induce hepatocyte apoptosis [21]. Thus, we next examined the effect of CoPP on the apoptotic death of hepatocytes in DDC-fed mice. TdT-mediated dUTP nick end labeling (TUNEL) assay revealed that DDC feeding largely increased the number of TUNEL-stained cells in the liver (Figure 4A,B). However, administration of CoPP decreased DDC-diet-induced hepatocyte apoptosis (Figure 4A,B). In addition, elevated protein levels of cleaved caspase-3 and cleaved poly(ADP-ribose) polymerase-1 (PARP-1) after DDC feeding were also reduced by CoPP (Figure 4C,D).

### 2.4. Induction of HO-1 by CoPP Suppressed Inflammatory Responses in DDC-Fed Mice

**Figure 5:**
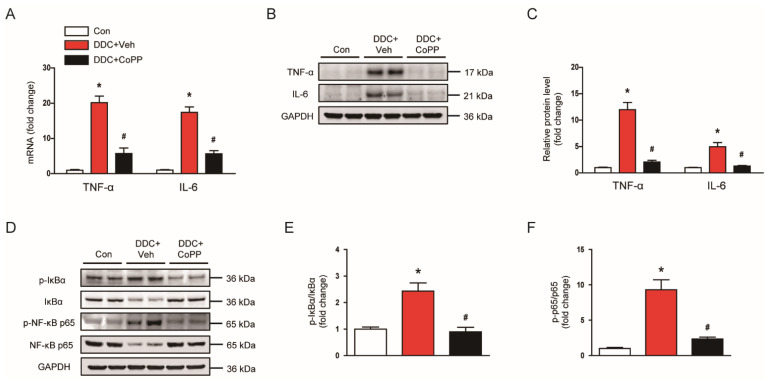
Effects of CoPP on cytokine production and the nuclear factor-κB (NF-κB) signaling pathway in DDC-fed mice. (**A**) The mRNA levels of tumor necrosis factor-α (TNF-α) and interleukin 6 (IL-6) in liver tissues. (**B**) Western blotting of TNF-α and IL-6 in liver tissues. (**C**) Quantification of Western blots for TNF-α and IL-6. (**D**) Western blotting of p-IκBα and p-NF-κB p65 in liver tissues. (**E**) Quantification of Western blot for p-IκBα. (**F**) Quantification of Western blot for p-NF-κB p65. n = 8 per group. * *p* < 0.05 vs. Con. ^#^ *p* < 0.05 vs. DDC + Veh.

Oxidative stress and hepatocyte apoptosis can induce inflammation and fibrosis in cholestatic liver diseases [20,21]. Therefore, we evaluated the effect of CoPP on inflammatory responses. DDC-fed mice exhibited increased mRNA expression of tumor necrosis factor-α (TNF-α) and interleukin 6 (IL-6) in the liver (Figure 5A). Western blot analysis confirmed the increased expression of TNF-α and IL-6 proteins (Figure 5B,C). However, these changes were significantly alleviated by CoPP (Figure 5A–C). Nuclear factor-κB (NF-κB) is an essential transcription factor modulating the expression of various inflammatory genes [22]. DDC feeding led to an increase in phosphorylated forms of IκBα and NF-κB p65 in the liver (Figure 5D–F). Administration of CoPP significantly inhibited the activation of the NF-κB signaling cascade (Figure 5D–F).

**Figure 6:**
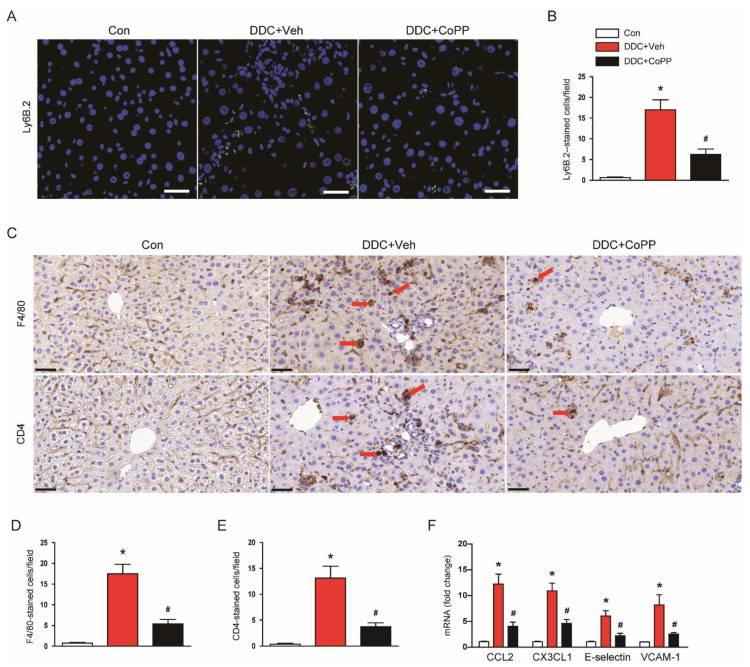
Effect of CoPP on immune cell accumulation in DDC-fed mice. (**A**) Immunofluorescent staining of liver tissues for Ly6B.2. Scale bar = 40 μm. (**B**) Number of Ly6B.2-stained cells. (**C**) Immunohistochemical staining of liver tissues for F4/80 or CD4. Red arrows indicate positively stained cells. Scale bar = 100 μm. (**D**) Number of F4/80-stained cells. (**E**) Number of CD4-stained cells. (**F**) mRNA levels of C–C motif chemokine ligand 2 (CCL2), C–X3–C motif chemokine ligand 1 (CX3CL1), E-selectin, and vascular cell adhesion molecule-1 (VCAM-1) in liver tissues. n = 8 per group. * *p* < 0.05 vs. Con. ^#^ *p* < 0.05 vs. DDC + Veh.

It was found that inflammatory cells were infiltrated into the liver tissue in DDC-fed mice, aggravating inflammatory responses [23,24]. Immunofluorescent staining for Ly6B.2 showed that the number of Ly6B.2^+^ neutrophils was increased after DDC feeding, which was reduced by CoPP (Figure 6A,B). In addition, immunohistochemical staining also revealed that the administration of CoPP inhibited the DDC-diet-induced accumulation of F4/80^+^ macrophages and CD4^+^ T cells in the liver (Figure 6C–E). Chemokines and vascular adhesion molecules promote the infiltration of inflammatory cells into tissues [25,26]. Thus, we also examined the expression of several key molecules. DDC feeding increased the mRNA expression of C–C motif chemokine ligand 2 (CCL2), C–X3–C motif chemokine ligand 1 (CX3CL1), E-selectin, and vascular cell adhesion molecule-1 (VCAM-1) (Figure 6F). These changes were significantly inhibited by CoPP (Figure 6F).

### 2.5. Induction of HO-1 by CoPP Inhibited the Fibrotic Process in DDC-Fed Mice

**Figure 7:**
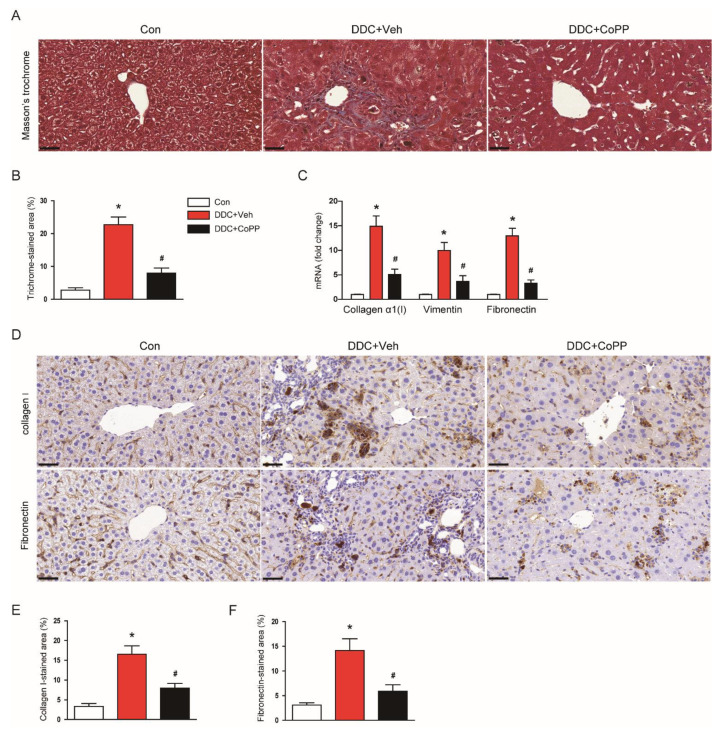
Effect of CoPP on DDC-diet-induced fibrosis. (**A**) Masson’s trichrome staining of liver tissues. Scale bar = 40 μm. (**B**) Percentage of trichrome-stained area. (**C**) mRNA levels of collagen α1(I), vimentin, and fibronectin in liver tissues. (**D**) Immunohistochemical staining of liver tissues for collagen I or fibronectin. Scale bar = 100 μm. (**E**) Percentage of collagen-I-stained area. (**F**) Percentage of fibronectin-stained area. n = 8 per group. * *p* < 0.05 vs. Con. ^#^ *p* < 0.05 vs. DDC + Veh.

We next investigated the effect of CoPP on the DDC-diet-induced fibrotic process. Masson’s trichrome staining showed marked fibrosis in the livers of DDC-fed mice (Figure 7A,B). DDC-fed mice exhibited increased mRNA expression of collagen α1(I), vimentin, and fibronectin in the liver (Figure 7C). Immunohistochemical staining also revealed that the percentage of the area stained with collagen I or fibronectin was markedly increased after DDC feeding (Figure 7D–F). However, the administration of CoPP significantly attenuated DDC-diet-induced liver fibrosis (Figure 7A–F).

### 2.6. Induction of HO-1 by CoPP Suppressed Myofibroblasts and Phosphorylation of Smad2/3 in DDC-Fed Mice

**Figure 8:**
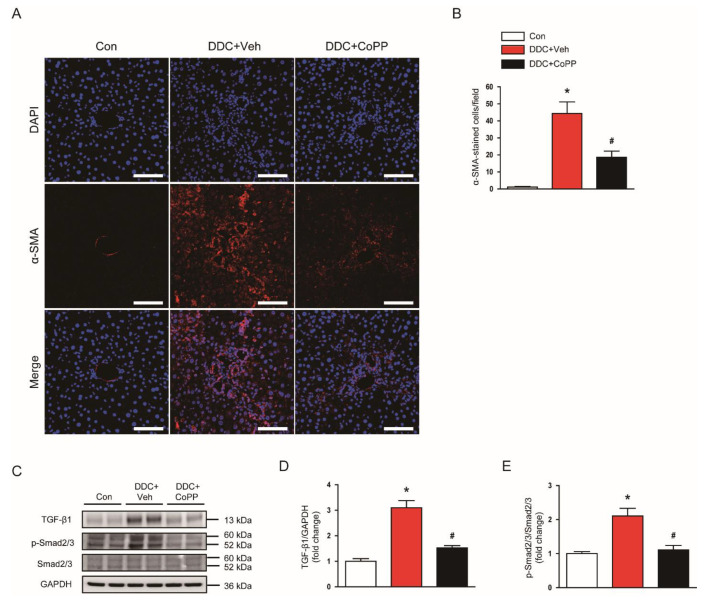
Effects of CoPP on myofibroblast accumulation and the transforming growth factor-β1 (TGF-β1) signaling cascade in DDC-fed mice. (**A**) Immunofluorescent staining of liver tissues for α-smooth muscle actin (α-SMA). Scale bar = 50 μm. (**B**) Number of α-SMA-stained cells. (**C**) Western blotting of TGF-β1 and p-Smad2/3 in liver tissues. (**D**) Quantification of Western blot for TGF-β1 (**E**) Quantification of Western blot for p-Smad2/3. n = 8 per group. * *p* < 0.05 vs. Con. ^#^ *p* < 0.05 vs. DDC + Veh.

Myofibroblasts secrete extracellular matrix proteins and contribute to tissue fibrosis [27,28]. Immunofluorescent staining for α-smooth muscle actin (α-SMA)—a marker of myofibroblasts [29]—showed that the number of α-SMA-stained cells was largely increased in the livers of DDC-fed mice (Figure 8A,B). Administration of CoPP significantly decreased the number of α-SMA^+^ myofibroblasts (Figure 8A,B).

The transforming growth factor-β1 (TGF-β1)/Smad2/3 intracellular pathway induces the expression of fibrosis-related genes [30]. Thus, we examined the effects of CoPP on TGF-β1 expression and Smad2/3 activation. DDC feeding resulted in increased expression of TGF-β1 and phosphorylated Smad2/3 in the liver, which was significantly inhibited by CoPP (Figure 8C–E).

## 3. Discussion

In the present study, we demonstrated that the pharmacological induction of HO-1 by CoPP ameliorated cholestatic liver injury and fibrosis by suppressing oxidative stress, hepatocyte apoptosis, and inflammation.

It is essential to understand the pathophysiology of cholestatic liver diseases in order to develop novel medical therapies. However, the pathogenic mechanisms are complex and incompletely elucidated [31]. Among potential pathogenic mechanisms, oxidative stress is considered to be a key contributor to cholestatic liver diseases [3,4]. Therefore, we hypothesized that the activation of the antioxidant system might be a useful therapeutic strategy for treating the disease, prompting us to investigate the effects of HO-1 induction in an animal model of cholestatic liver injury. Because the DDC diet model is the most popular model to study this disease [15,16], we used this animal model in the present study.

HO-1 is a potent antioxidant enzyme, and its expression is highly induced under stressful conditions [12]. Growing evidence suggests that the induction of HO-1 may be a potential therapeutic approach for treating various inflammatory diseases, such as gastrointestinal [12], cardiovascular [13], or respiratory diseases [14]. HO-1-knockout mice exhibited increased renal inflammation and fibrosis, with enhanced epithelial–mesenchymal transition of tubular epithelial cells, after unilateral ureteral obstruction [32]. The pharmacological induction of HO-1 by hemin inhibited the profibrotic effects of TGF-β in renal tubular epithelial cells [33]. Barikbin et al. investigated the effects of HO-1 induction in Mdr2-knockout mice [34]; they found that the administration of CoPP effectively suppressed liver inflammation and fibrosis in the genetic animal model of chronic liver disease [34]. Recent studies have also shown that inflammatory and fibrotic changes in fatty liver disease were effectively attenuated by HO-1 induction [35,36]. In addition, pharmacological or genetic induction of HO-1 suppressed carbon-tetrachloride-induced liver fibrosis in rodents [37,38]. However, the potential effect of HO-1 induction against cholestatic liver injury and fibrosis has not yet been investigated. In this study, we observed that DDC feeding largely increased HO-1 expression in the liver, indicating that HO-1 induction is an adaptive defense mechanism for protection against cholestatic liver injury. However, the degree of HO-1 induction seems to be insufficient to inhibit liver damage and fibrosis. Interestingly, further enhancement of HO-1 induction by CoPP effectively suppresses DDC-diet-induced cholestatic liver injury.

Current evidence suggests that oxidative stress induces cholestasis by inhibiting hepatobiliary secretory function [3]. In this study, we showed that the induction of HO-1 attenuated DDC-diet-induced oxidative stress, as evidenced by reduced expression of markers of oxidative DNA damage and lipid peroxidation. The inhibition of oxidative stress was accompanied by decreased levels of serum AST, ALT, ALP, and total bilirubin, indicating a reduction in hepatocyte damage and cholestasis. Cholestasis leads to the accumulation of bile acids in serum and in hepatocytes [1]. Bile-acid-induced apoptosis is considered to be a major cause of hepatocyte damage [39]. In this study, we found that hepatocyte apoptosis was increased after DDC feeding, and was reduced by HO-1 induction. In agreement with our data, previous studies have shown that HO-1 exerts anti-apoptotic action in various types of cells [40,41,42]. Taken together, these results suggest that the suppression of oxidative stress by HO-1 induction led to improvement of cholestasis, resulting in a reduction of hepatocyte apoptosis.

Oxidative stress can also lead to liver inflammation, as well as hepatocyte apoptosis—both of which are central events in liver fibrosis [3,4]. Previous studies have shown that DDC feeding results in a marked production of cytokines and excessive infiltration of inflammatory cells into the liver [23,24]. In this study, DDC feeding increased hepatic levels of cytokines. This inflammatory response was accompanied by activation of the NF-κB signaling cascade. However, the administration of CoPP significantly attenuated the increased production of cytokines, along with inhibition of the NF-κB pathway. Increased accumulation of neutrophils, macrophages, and CD4^+^ T cells was also inhibited by CoPP. Chemokines and vascular adhesion molecules promote inflammatory cell infiltration [25,26]. We found that upregulation of these molecules was significantly inhibited by CoPP. Altogether, these data suggest that the induction of HO-1 attenuated cytokine production and inflammatory cell infiltration by suppressing the NF-κB signaling cascade, and via the downregulation of chemokines and vascular adhesion molecules. Consistent with our results, recent studies have reported that the pharmacological induction of HO-1 by hemin or CoPP inhibits inflammatory responses in various types of tissues [43,44,45].

Oxidative stress, hepatocyte apoptosis, and inflammation are critically involved in the pathophysiology of liver fibrosis [46]. In this study, we found that the induction of HO-1 effectively inhibited liver fibrosis in DDC-fed mice, as reflected by decreased staining with Masson’s trichrome and reduced expression of fibrosis-related genes. Myofibroblasts are cells that synthesize and secrete extracellular matrices into the tissue, and are characterized by their expression of α-SMA [27,28,29]. A previous study has reported that DDC feeding markedly increased the number of α-SMA^+^ myofibroblasts in the liver [47]. This result was confirmed by immunofluorescent staining in the present study. The administration of CoPP significantly inhibited the DDC-diet-induced accumulation of myofibroblasts. Given that myofibroblasts are a key player in fibrosis, their suppression by HO-1 induction is an important contributor to the antifibrotic action of CoPP. TGF-β1 plays a crucial role in the development of fibrotic diseases [30]. Binding of TGF-β1 to its receptors leads to an increase in phosphorylated Smad2 and Smad3, which form a heteromeric complex with Smad4. The TGF-β/Smad signaling cascade regulates the expression of fibrosis-associated genes [30]. In this study, we found that HO-1 induction significantly inhibited TGF-β1 expression and Smad2/3 activation in the livers of DDC-fed mice, indicating the suppressive action of HO-1 on the TGF-β/Smad pathway.

## 4. Materials and Methods

### 4.1. Animals Procedures

Male C57BL/6N mice (7 weeks old, n = 24) were purchased from HyoSung Science Inc. (Daegu, Korea). These mice were maintained at 20–24 °C and 60–70% humidity under a 12-h light/dark cycle, and were allowed to acclimatize to their housing conditions for 1 week. The mice were arbitrarily separated into 3 groups (n = 8) as follows: (a) vehicle-treated control group (Con): C57BL/6N mice were fed with a normal chow diet for 4 weeks; (b) DDC-fed group (DDC + Veh): C57BL/6N mice were fed with a diet containing 0.1% DDC (RaonBio Inc., Yongin, Korea) for 4 weeks; (c) DDC-fed group treated with CoPP (DDC + CoPP): C57BL/6N mice were fed with a diet containing 0.1% DDC, and were given an intraperitoneal injection of CoPP (5 mg/kg; Sigma-Aldrich, St. Louis, MO, USA) twice a week for 4 weeks. CoPP was dissolved in dimethyl sulfoxide and then diluted with normal saline. The Con group and the DDC + Veh group were intraperitoneally injected with an equal volume of the vehicle twice a week for 4 weeks. The dose of CoPP and duration of treatment were determined based on previous studies [34,48,49]. All mice were euthanized after 4 weeks of treatment, and blood and liver samples were rapidly collected for biochemical and histological analysis. All animal care and experimental protocols were performed in accordance with the Institutional Animal Care and Use Committee of the Daegu Catholic University Medical Center (Approval number: DCIAFCR-210112-18-Y, approval date: 12 January 2021).

### 4.2. Biochemical Analysis

Serum AST, ALT, ALP, and total bilirubin levels were measured using a 7020 automatic analyzer (Hitachi, Osaka, Japan). MDA levels were analyzed using a Lipid Peroxidation Assay Kit (Sigma-Aldrich, St. Louis, MO, USA), according to the manufacturer’s protocol.

### 4.3. Histological Analysis and Immunohistochemistry

Liver tissues were fixed in 10% formalin and dehydrated. Paraffin-embedded sections (4 μm) were stained with H&E or Masson’s trichrome. For immunohistochemical staining, the sections were incubated with a primary antibody against 4-HNE (1:100), F4/80 (1:100), CD4 (1:500), collagen I (1:100), or fibronectin (1:100). These antibodies were purchased from Abcam (Cambridge, UK), except for F4/80 (Santa Cruz Biotechnology Inc., Dallas, TX, USA). After washing, the sections were probed with horseradish peroxidase (HRP)-conjugated goat anti-rabbit secondary antibody (1:1000; Abcam, Cambridge, UK). Images were captured using a confocal microscope (Nikon, Tokyo, Japan). The percentage of positively stained area was analyzed in 5 randomly selected fields per liver sample, using i-Solution DT software (IMT i-Solution Inc., Coquitlam, BC, Canada). The number of F4/80 or CD4-stained cells was counted in 5 randomly selected fields per liver sample.

### 4.4. Immunofluorescent Staining

For immunofluorescent staining, liver sections were blocked in a blocking buffer (5% bovine serum albumin in phosphate-buffered saline). After blocking, the sections were incubated with a primary antibody against 8-OHdG (1:100; Santa Cruz Biotechnology Inc., Dallas, TX, USA), Ly6B.2 (1:100; Abcam, Cambridge, UK), or α-SMA (1:100; Sigma-Aldrich, St. Louis, MO, USA). After washing, the sections were probed with a secondary antibody conjugated with Alexa Fluor 488 or Alexa Fluor 555 (Invitrogen, Carlsbad, CA, USA), and then counterstained with 4′,6-diamidino-2-phenylindole (DAPI). The number of positively stained cells was counted in 5 arbitrarily chosen fields per liver sample.

### 4.5. TUNEL Assay

TUNEL assay was performed using a commercial kit (Roche Diagnostics, Indianapolis, IN, USA), according to the manufacturer′s instructions. Nuclei were counterstained with DAPI. The number of TUNEL-stained cells was counted in 5 arbitrarily chosen fields per liver sample.

### 4.6. Western Blot Analysis

Liver tissues were lysed in a lysis buffer (Sigma-Aldrich, St. Louis, MO, USA). Protein concentrations were determined using a BCA protein assay kit (Bio-Rad Laboratories, Hercules, CA, USA). Protein samples (8–10 μg) were loaded onto precast gradient polyacrylamide gels (Thermo Fisher Scientific, Waltham, MA, USA) and then transferred to a nitrocellulose membrane. The membranes were incubated with one of the following primary antibodies: anti-HO-1 (1:1000; Invitrogen, Carlsbad, CA, USA), anti-caspase-3 (1:1000; Cell Signaling, Danvers, MA, USA), anti-PARP-1 (1:1000; Cell Signaling, Danvers, MA, USA), anti-TNF-α (1:1000; Abcam, Cambridge, UK), anti-IL-6 (1:1000; Abcam, Cambridge, UK), anti-IκBα (1:1000; Cell Signaling, Danvers, MA, USA), anti-p-IκBα (1:1000; Cell Signaling, Danvers, MA, USA), anti-NF-κB p65 (1:1000; Cell Signaling, Danvers, MA, USA), anti-p-NF-κB p65 (1:1000; Cell Signaling, Danvers, MA, USA), anti-TGF-β1 (1:1000; Abcam, Cambridge, UK), anti-Smad2/3 (1:1000; Cell Signaling, Danvers, MA, USA), anti-p-Smad2/3 (1:1000; Cell Signaling, Danvers, MA, USA), or anti-glyceraldehyde-3-phosphate dehydrogenase (GAPDH; 1:3000; Cell Signaling, Danvers, MA, USA). Then, the membranes were probed with an HRP-conjugated secondary antibody. The signal intensities were measured using the iBright™ CL1500 Imaging System (Thermo Fisher Scientific, Waltham, MA, USA).

### 4.7. Real-Time Reverse Transcription Polymerase Chain Reaction (RT-PCR)

RNA was extracted from liver tissues using the TRIzol reagent (Sigma-Aldrich, St. Louis, MO, USA). First-strand cDNA was synthesized from RNA using the RNA to cDNA EcoDry™ Premix kit (TaKaRa, Tokyo, Japan), according to the manufacturer′s instructions. Amplification of cDNA was performed using SYBR Green dye with the Thermal Cycler Dice Real Time System III (TaKaRa, Tokyo, Japan). The primer sequences used in this study are listed in Table 1. GAPDH was used as a reference gene.

### 4.8. Statistical Analysis

Data are presented as mean ± standard error of the mean (SEM). Statistical analyses were performed using the one-way analysis of variance (ANOVA) with Bonferroni’s post hoc tests. A *p*-value less than 0.05 was considered to represent statistical significance.

## 5. Conclusions

In conclusion, our data suggest that the administration of CoPP effectively ameliorates liver injury and fibrosis along with HO-1 induction in a murine model of cholestatic liver disease. These effects were associated with the suppression of oxidative stress, hepatocyte apoptosis, and inflammation. HO-1 induction might be a useful therapeutic approach for treating this disease.

## Figures and Tables

**Table 1:** List of primers used in this study.

Gene	Primer Sequence (5′→3′)
HO-1 ^1^	Sense: TCAAGGCCTCAGACAAATCC Antisense: ACAACCAGTGAGTGGAGCCT
TNF-α ^2^	Sense: GACGTGGAACTGGCAGAAGAG Antisense: CCGCCTGGAGTTCTGGAA
IL-6 ^3^	Sense: CCAGAGATACAAAGAAATGATGG Antisense: ACTCCAGAAGACCAGAGGAAAT
CCL2 ^4^	Sense: GGGCCTGCTGTTCACAGTT Antisense: CCAGCCTACTCATTGGGAT
CX3CL1 ^5^	Sense: ATGACCTCACGAATCCCAGTG Antisense: CCGCCTCAAAACTTCCAATGC
E-selectin	Sense: AGCTACCCATGGAACACGAC Antisense: ACGCAAGTTCTCCAGCTGTT
VCAM-1 ^6^	Sense: CCCAGGTGGAGGTCTACTCA Antisense: CAGGATTTTGGGAGCTGGTA
collagen α1(I)	Sense: GAGTGAGGCCACGCATGA Antisense: AGCCGGAGGTCCACAAAG
vimentin	Sense: GATCGATGTGGACGTTTCCAA Antisense: GTTGGCAGCCTCAGAGAGGT
fibronectin	Sense: CGAGGTGACAGAGACCACAA Antisense: CTGGAGTCAAGCCAGACACA
GAPDH ^7^	Sense: ACTCCACTCACGGCAAATTC Antisense: TCTCCATGGTGGTGAAGACA

^1^ Heme oxygenase-1. ^2^ Tumor necrosis factor-α. ^3^ Interleukin-6. ^4^ C–C motif chemokine ligand 2. ^5^ C–X3–C motif chemokine ligand 1. ^6^ Vascular cell adhesion molecule-1. ^7^ Glyceraldehyde-3-phosphate dehydrogenase.

## Data Availability

Data are contained within the article.
